# Biofertilizers and Biocontrol Agents for Agriculture: How to Identify and Develop New Potent Microbial Strains and Traits

**DOI:** 10.3390/microorganisms9040817

**Published:** 2021-04-13

**Authors:** Anna Maria Pirttilä, Habibollah Mohammad Parast Tabas, Namrata Baruah, Janne J. Koskimäki

**Affiliations:** Ecology and Genetics, University of Oulu, FIN-90014 Oulu, Finland; Habibollah.MohammadParastTabas@student.oulu.fi (H.M.P.T.); namrata.baruah@oulu.fi (N.B.); janne.koskimaki@oulu.fi (J.J.K.)

**Keywords:** genome mining, plant–microbe interactions, microbe–microbe interactions, bacteriophage, microbiome engineering

## Abstract

Microbiological tools, biofertilizers, and biocontrol agents, which are bacteria and fungi capable of providing beneficial outcomes in crop plant growth and health, have been developed for several decades. Currently we have a selection of strains available as products for agriculture, predominantly based on plant-growth-promoting rhizobacteria (PGPR), soil, epiphytic, and mycorrhizal fungi, each having specific challenges in their production and use, with the main one being inconsistency of field performance. With the growing global concern about pollution, greenhouse gas accumulation, and increased need for plant-based foods, the demand for biofertilizers and biocontrol agents is expected to grow. What are the prospects of finding solutions to the challenges on existing tools? The inconsistent field performance could be overcome by using combinations of several different types of microbial strains, consisting various members of the full plant microbiome. However, a thorough understanding of each microbiological tool, microbial communities, and their mechanisms of action must precede the product development. In this review, we offer a brief overview of the available tools and consider various techniques and approaches that can produce information on new beneficial traits in biofertilizer and biocontrol strains. We also discuss innovative ideas on how and where to identify efficient new members for the biofertilizer and biocontrol strain family.

## 1. Introduction

The UN Food and Agriculture Organization (FAO) estimates that farmers will have to produce 70% more food by 2050 to meet the global needs of the predicted population of 9-billion people on earth (www.fao.org). A recent development is the popularity of plant-based food over meat and dairy production to balance the global greenhouse gas budget [1]. However, there is a simultaneous increased global concern on pollution by inorganic residuals of fertilizers, as well as effects of synthetic plant protection compounds on human and animal health [2,3,4].

Chemical fertilizers play an important role in fulfilling the continuously increasing food demand of the world population. The three major types of commercial fertilizers, nitrogen (N), phosphate (P), and potassium (K), are used to achieve maximum yields in crop production [5]. However, increased agricultural use of chemical fertilizers causes harmful impacts on ecosystems. Due to insufficient uptake of chemical fertilizers by plants, they eventually enter the water bodies through leaching, where they cause eutrophication. Furthermore, they can have various harmful effects on soils, resulting in depletion of water holding capacity and unbalanced soil fertility [6]. Besides being costly, they increase greenhouse gas emissions due to fossil fuel use in their production. For a long time, there has been a need to develop alternative, low cost, effective, and ecofriendly fertilizers, which work without disturbing the nature [7].

Another serious problem is the extensive use of pesticides, many of which are harmful not only for humans, but also for animals, such as pollinators [8]. Furthermore, pesticides may alter the composition of plant-associated microbial communities in the soil [9]. There have been attempts to reduce the use of toxic pesticides; for example, the European Union (EU) promotes use of less harmful chemicals in agriculture through the European Pesticide Regulation (EC) No. 1107/2009. Whereas the European Pesticide Regulation is the strictest one drafted among the four major agriculture producers, European Union, United States, China, and Brazil [10], even this document contains loopholes, which result in health hazards posed by pesticides for humans within the EU [11]. The reason for a high tolerance of toxic pesticides worldwide might be that a tight regulation of agrochemicals creates challenges in plant production and can result in crop-yield reductions. Furthermore, climatic change poses a threat of unpredictable yields due to increased abiotic and biotic stresses on crop plants [12,13]. Therefore, the search for environmentally friendly alternatives is becoming imperative.

Microbiological tools, namely biofertilizers and biocontrol agents, which are bacteria and fungi capable of providing beneficial outcomes in plant growth and health, respectively, have been developed for several decades. Biofertilizers have a great potential to improve crop yields through environmentally friendly mechanisms [7]. A biofertilizer is defined as a product which contains living microorganisms that, when applied to soil, seeds, or surfaces of plant, colonize the rhizosphere or the plant internal tissues and induce plant growth. Biofertilizers are typically bacteria or fungi capable of nitrogen fixation, phosphate solubilization, sulfur oxidization, plant hormone production, or decomposition of organic compounds [14]. For example, *Pseudomonas fluorescens* K-34 produces organic acids, potentially responsible for phosphate release for the plant. *P. fluorescens* K–34, *P. fluorescens* 1773/K, *P. trivalis* BIHB 745, and *Bacillus circulans* are also capable of producing the plant hormone indole acetic acid (IAA) [15]. Overall, biofertilizers carry out nutrient cycling and ensure optimal growth and development of crops [16].

The microbial inoculants potentially replacing harmful pesticides are called biocontrol agents. Biological control, by definition, provides a non-chemical method for management of plant diseases by using other living entities, such as microorganisms. The biocontrol capacity of a microbe can result from production of antibiotic compounds, or enzymes capable of fungal cell wall lysis, depletion of iron from the rhizosphere, induced systemic resistance, and competition for niches with pathogens within the rhizosphere [15]. Production of one or more antibiotics is a mechanism most commonly associated with biocontrol ability. A number of biocontrol strains can also produce antifungal enzymes, for example chitinases, *β*1,3-glucanases, proteases, or lipases, with the capacity to lyse fungal cells. Synthesis of low-molecular mass siderophores that chelate iron in the soil near roots can inhibit the proliferation of fungal pathogens [17]. For example, *Pseudomonas trivalis* strain BIHB 745 can produce siderophores [15], and the siderophores pyochelin and pyoverdine have been identified in *P. fluorescens* [18]. Many biocontrol strains can protect the host plant by out-competing phytopathogens for nutrients. They help the plant also by colonizing niches in the rhizosphere and preventing pathogens from infecting the plant [19].

In general, microbial inoculants are promising tools for sustainable agriculture, because, optimally, they can both support the health of the plant along with promoting plant growth and enhancing nutrient availability and uptake [20]. In this paper, we briefly review the microbiological tools that have been developed and consider their formulation and current status for application in crop production. We then discuss approaches potentially revealing new traits of biofertilizers and biocontrol agents, and how to screen for new strains usable in modern environmentally friendly agriculture.

## 2. Microorganisms Used in Biocontrol and Biofertilization

### 2.1. Plant-Growth-Promoting Rhizobacteria (PGPR)

The most common biofertilizers and biocontrol agents currently in use belong to a group known as plant-growth-promoting rhizobacteria (PGPR) [21,22]. PGPR colonize the rhizosphere of many plant species, where they induce beneficial effects for the host, for example, increased plant growth and reduced susceptibility to diseases caused by plant pathogens, such as nematodes, fungi, bacteria, and viruses [19]. The majority of the most-well known PGPR belong to the genera *Alcaligenes*, *Arthrobacter*, *Azospirillum*, *Azotobacter*, *Bacillus*, *Burkholderia. Enterobacter*, *Klebsiella*, *Pseudomonas*, *Rhizobium*, and *Serratia* [19]. Benefits of PGPR can include increased seed germination rate, root growth, yield, leaf area, chlorophyll content, nutrient uptake, protein content, hydraulic activity, tolerance to abiotic stress, shoot and root weights, and delayed senescence [23].

PGPR are, thus, often employed as biofertilizers. For example, *Azotobacter chroococcum* and *A. vinelandii* are used as nitrogen fertilizers worldwide. Strains of *Bacillus megaterium*, *B. amyloliquefaciens* IT45, and *Pseudomonas fluorescens* are used for phosphorus fertilization. Providing potassium nutrition for crops, there are biofertilizer products based on *Frateuria aurantia* (Table S1). For zinc, sulfur, and silicate fertilization, there are strains such as *Thiobacillus thiooxidans*, *Delfia acidovorans*, and *Bacillus* spp. used as products mainly in Asia. Furthermore, there are >10 products with undefined mechanism (apart from “plant growth promotion”) consisting of 1–30 strains of PGPR [24].

Many *Pseudomonas* species produce a broad spectrum of antibiotics, including pyoluteorin (PLT), pyrrolnitrin (PRN), 2,4-diacetylphloroglucinol, and hydrogen cyanide, and the strain *P. fluorescens* ZX produces and secrets lytic enzymes [18]. Currently, there are several PGPR-derived products available for biocontrol based on, e.g., *Bacillus* strains, *Pseudomonas* strains, and *Streptomyces griseoviridis* (Supplementary Table S1) [21,24]. For example, *P. fluorescens* A506 controls fire blight on pome fruits by competing with the pathogen *Erwinia amylovora* [25]. On the other hand, *P. chlororaphis* is effective on barley net blotch (*Drechslera teres*), barley leaf stripe (*Drechslera graminae*), and *Fusarium* pathogens [26,27]. *B. subtilis* increases growth and salt tolerance in *Arabidopsis* [28] and prevents the mummy berry fungus *Monilinia vaccinii-corymbosi* in blueberry [29], whereas *B. amyloliquefaciens* controls bottom rot on lettuce [30]. *S. griseoviridis* K61 is effective against many fungal pathogens, such as *Fusarium oxysporum* f.sp. *lycopersici* and *Verticillium dahliae*, which are responsible of vascular wilt of tomato and *Verticillium* wilt in many crop plants [31].

### 2.2. Rhizobia

Rhizobia are among the oldest agricultural tools, as the industrial production of rhizobia started already at the end of the 19th century. The nitrogen-fixing alpha-protebacterial genera of *Agrobacterium*, *Allorhizobium*, *Azorhizobium*, *Bradyrhizobium*, *Mesorhizobium*, *Rhizobium*, *Sinorhizobium*, *Devosia*, *Methylobacterium*, *Ochrobactrum*, and *Phyllobacterium*, as well as beta-proteobacterial *Burkholderia* and *Cupriavidus,* can all form nodules with legumes as the host plant [32]. Whereas their agricultural use is rather limited to the leguminous crop plants, they are responsible of as much as 200 to 300 kg of fixed nitrogen/ha/crop. Furthermore, the nitrogen-fixing cyanobacterial symbiosis with *Azolla* is widely used as green manures in rice cultivation [32].

### 2.3. Mycorrhizal Fungi

Besides PGPR, mycorrhizal fungi are among the most used biofertilizers globally [33]. They extend the root system of the host plant and help the crop plants in uptake of water and nutrients, specifically phosphorus; reduce abiotic stress; and act in biocontrol against root-damaging pathogens of the genera *Fusarium*, *Pythium*, and *Phytophthora*, as well as nematodes [34,35,36]. Mycorrhizal fungi can improve the food-quality properties of crop plants by increasing antioxidant and vitamin contents of edible parts [37,38]. Furthermore, they improve the physical properties of the soil by modifying the soil structure. For example, the hyphae can induce entanglement of soil particles with each other to create macroaggregates [39]. The most common type used to improve agricultural crops is arbuscular mycorrhizal fungi (AMF), which penetrate the root cortical cells of a host plant and form highly branched structures, arbuscules [34]. The glycoprotein of AMF, glomalin, is an important component of the mycorrhizal soil that mitigates formation of gas-water interfaces by reducing macroaggregate disruption during wetting and drying, and by preventing movement of water into soil pores [39]. The AMF are favored in improving agricultural crops, because they are naturally found in over 90% of plant species, and many agricultural practices, such as tillage and fertilization, can reduce abundance of AMF in the fields [40]. As agricultural additives, they have been shown to increase grain yields depending on soil pH, plant species, and cultivar [41]. For example, AMF strains can increase tomato yields by 26% and carrot yields by 300% by protecting host plants against nematodes [42]. A meta-analysis suggests that C4 grasses, non-legume, and woody plants respond better to AMF than legumes and C3 grasses, especially when the soil community is complex and limited in phosphorus rather than nitrogen. Hoeksema et al. state that, besides the specifics of the plant–mycorrhiza interaction, the outcome of mycorrhizal inoculation depends on fertility and biocomplexity of the soil, and environmental conditions, such as salinity and pH [43]. For better predictivity of mycorrhizal inoculation, there is a MycoDB database consisting of meta-analysis data spanning > 10 years on plant productivity responses to mycorrhizal applications [44].

Regardless of promising results with mycorrhizal inoculants, there are many technical challenges in their production. The up-scaling of mass production of mycorrhizal fungi is often hard, as some strains of the mutualistic symbionts are not cultivable in axenic conditions. With existing protocols, the mycorrhizal inocula may not be pure but contain fungal pathogens, therefore molecular tests are needed for analysis of the inocula composition [33]. However, there are several biofertilizer and biocontrol products of mycorrhizal fungi available, based mainly on strains of *Glomus iranicum*, to improve crop absorption of water and nutrients, as well as tolerance towards nematodes. Other mycorrhizal inoculants contain spores of *Rhizofagus irregularis*, *Funneliformis mossae*, and *Claroideoglomus etunicatum* for improved fruit production (Table S1) [45].

### 2.4. Endophytic Fungi

Regardless of numerous positive impacts on plants and potential in both biocontrol and biofertilization, endophytic fungi have been rarely developed as biotechnological tools in agriculture. One of the most promising and best studied endophytes is *Piriformospora indica*, which colonizes the roots of crop plants such as barley and corn [46,47,48]. The fungus can promote the uptake of phosphorus and sulfur, enhance production of biomass, and encourage early flowering and seed production [49,50]. *P. indica* helps the host plant to overcome abiotic stress, such as water, temperature, and salt stresses, and induces plant resistance towards toxins, heavy metals, insects, and pathogens [51]. The fungus thrives in the southern hemisphere, as the DNA and mRNA of *P. indica* were detected in the soil in UK only for a maximum of 15 months [52]. Overall, the effects of *P. indica* have been thoroughly studied and tested in as many as 150 different plant species [53].

The clavicipitaceous endophytes of grasses are an example of fungi that are used in biocontrol in agriculture [12]. The tall fescue has been inoculated with *Epichloë coenophiala* for enhanced pest tolerance [54,55], and ryegrass varieties inoculated with fungal endophytes are used in New Zealand and Australia with reduced pasture damage by insect herbivores [56,57]. However, the use of clavicipitaceous endophytes is currently limited to pasture grasses only [12].

### 2.5. Rhizospheric Fungi

There are also several soil-associated fungi used in agriculture for increasing crop health and fitness (Supplementary Table S1). For example, *Penicillium bilaiae* is a rhizospheric fungus that, like mycorrhizal fungi, helps the plant in phosphate acquisition [45,58]. Members of rhizospheric and epiphytic *Trichoderma* spp. are used in many products due to their capacity to reduce abiotic and biotic stress on host plants, for example, by controlling many plant pathogens and nematodes [33]. *Trichoderma viride* is antagonistic towards soil borne pathogens in genera *Fusarium*, *Sclerotium*, *Rhizoctonia*, and *Pythium*. *T. harzianum* can control the same fungal pathogens as *T. viride*, but also *Botrytis*, *Gaeumannomyces*, *Sclerotinia*, *Verticillium*, and wood-rot fungi. *T. harzianum* and *T. polysporum* are used together to increase the spectrum of activity against diseases caused by fungal pathogens [33], and *Trichoderma* spp. can also be used in combination with mycorrhizal fungi. Together with *Glomus intraradices*, the *T. atroviride* strain is capable of producing siderophores and auxin-like compounds that are able to increase the growth of zucchini, lettuce, pepper, melon, and tomato by 56–167% [59]. *T. harzianum* can also induce the growth, flowering, and secondary metabolism of host plants [60].

### 2.6. Mycoparasitic and Entomopathogenic Fungi

In addition to plant-associated fungi, there are several fungal products available based on mycoparasitism of plant pathogens. *Ampelomyces quisqalis* and *Fusarium proliferatum* are mycoparasites of fungi causing powdery and downy mildew on crop plants, respectively [33]. *Beauveria bassiana* is an entomopathogenic fungus used to control pathogenic insects, such as white flies, thrips, mites, aphids, and their various developmental stages infesting numerous crop plants [33,61]. Furthermore, *Chaetomium cupreum* protects plants from fungal diseases such as rust, early and late blight, leaf spot, and stem and tuber rot [62].

## 3. Formulation

A competent biocontrol or biofertilizer strain needs to be manufactured in a form that is easy to apply on crops. A good formulation is simple, low in cost, and fluently transported. Whether to manufacture the strain in liquid or solid form is an important choice to make, as it affects shelf life and application method on crops [63]. The viability of microbial biomass during the process is critical and a priority in the selection of the method. Other issues to be considered are adhesion and coverage of microbial cells on the target site, and microbial viability after application. Solid formulations include granules or microgranules, dusts, and wettable powders, whereas liquid formulations can be based in water, oil, or an emulsion [64], reviewed in detail by Bashan et al. [65]. Bacterial inoculants are manufactured in both solid and liquid forms. The best strains from the bioformulation point of view are Gram-positive sporulating bacteria, due to the high resistance of spores to various treatments. Similarly, sporulating fungi are often well suited for dry formulation, such as powder or granules [66,67]. However, several biofertilizer or biocontrol strains belong to Gram-negative bacteria, which complicates their bioformulation, as they are more sensitive to various environmental conditions, such as drought or heat [68]. Regardless, dry formulations of Gram-negative bacteria have been reported successful [69,70]. The formulation can be a bottleneck in development of a biocontrol or biofertilizer product. For example, preventing contamination of the product is a key aspect, as unwanted microbial cells can completely inactivate or change the properties of the strain. A simple and low-cost manufacturing process may be necessary, but formulating the strain in sterile conditions can significantly increase production costs [71].

## 4. Current Trends

**Figure 1:**
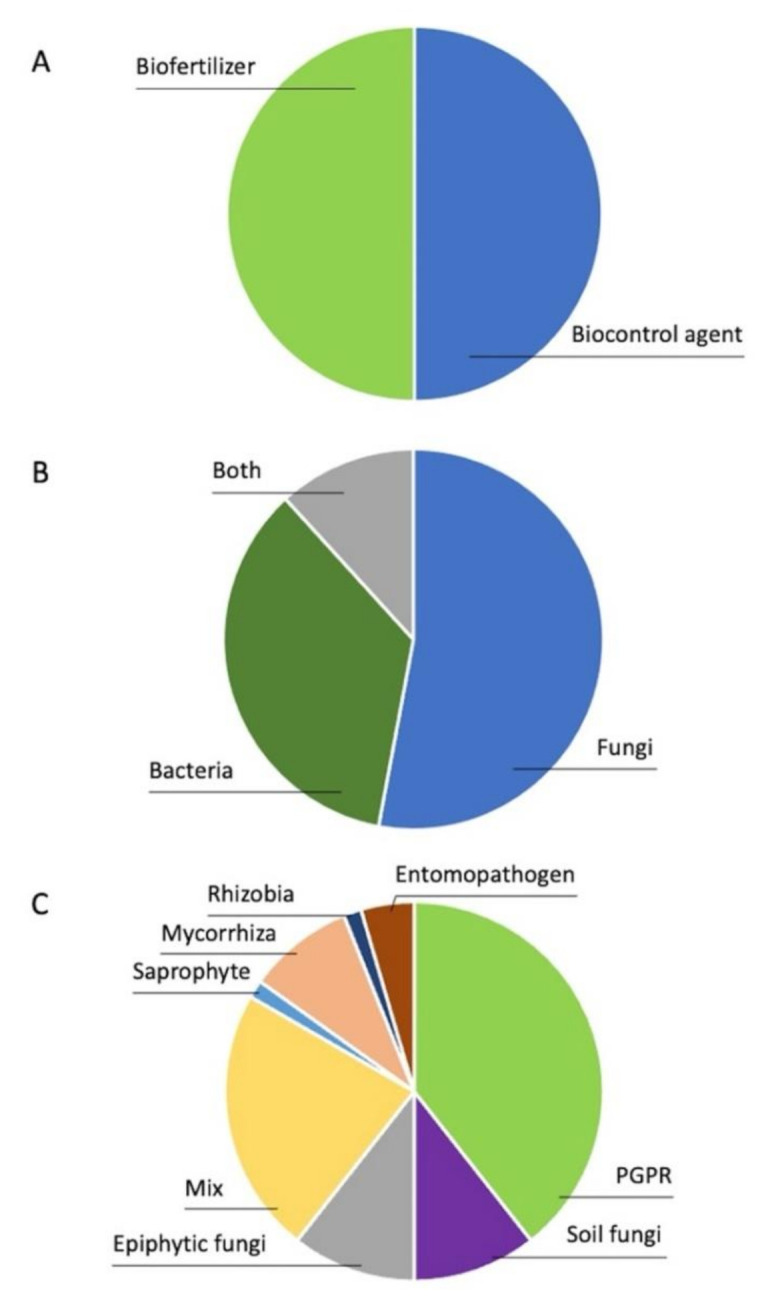
Microbiological tools, available in 2021 for agriculture (Supplementary Table S1) with published field test reports by (**A**) application, (**B**) organism, and (**C**) type.

A great majority of the microbiological tools currently available colonize the rhizosphere of a crop plant [45], consisting mainly of PGPR, epiphytic, mycorrhizal, and soil fungi (Figure 1). Consequently, their application often results in inconsistent field performances in agriculture. These variations can be associated with poor competence of inoculants in the field conditions, plant genotype, interactions with the existing plant-host microbiome, and environmental conditions. The soil ecosystem is highly variable, with localized microenvironments that are affected by changing temperatures and humidity. Soil microbial communities can have a crucial role in survival of the incoming microbes. Depending on soil quality, environmental conditions, and the interactions between soil microbiome, the inoculant, and the host plant, the biofertilizer or biocontrol agent can have varying rates of success in increasing the crop yield [63,72]. A constant search for new potent, stable strains and the effort for deeper understanding of mechanisms of action of biofertilizers and biocontrol agents will be the key for achieving dependable tools for improved and sustainable crop production.

## 5. How to Identify New Potent Microbial Traits

Currently, agriculture relies on a few microbial beneficial traits improving the growth of various plant crops. Biofertilizers typically help plant growth by fixing nitrogen, solubilizing phosphates, producing plant hormones, oxidizing sulfur, and decomposing organic compounds, whereas biocontrol agents induce systemic resistance, produce one or several antibiotic compounds or enzymes, deplete iron, and compete for niches with pathogens within the rhizosphere, all reviewed in detail several times in the past (see, e.g., References [17,73,74]). However, other yet-unexplored microbial traits beneficial for the host plant likely exist, typical for each strain. Furthermore, specific traits and concerted action of microbial communities can result in improved plant growth and stress tolerance, currently unstudied [75]. Next, we discuss the methods, studies, and approaches that can generate data on new microbial traits potentially important in the function of biofertilizers and biocontrol agents.

### 5.1. Genome Mining

Sequencing of genomes and analysis of gene functions could reveal new mechanisms on how microbes help plants thrive. The genome of the first commercially available biocontrol agent, *Bacillus amyloliquefaciens* GB03, was reported in 2014 [76], and the genome of *B. velezensis* FZB42, which is considered a model for plant-growth-promoting and biocontrol rhizobacteria, was sequenced already in 2007 [77]. Since then, numerous bacterial strains found or suggested effective in biocontrol or biological fertilization of various crop plants have been sequenced [21,78]. So far, genome mining of the strains has, however, focused on known traits, listed above. For example, the genome of *B. velezensis* FZB42 was reported to carry 13 gene clusters responsible for synthesis of predicted antimicrobial metabolites or volatile compounds [79]. Sequencing of several other biocontrol strains of *B. velezensis*, WRN014, 9912D, M75, RC 218, and CC09 have revealed antimicrobial clusters in their genomes [80], and biosynthesis genes of surfactin, bacillaene, fengycin, and bacillibactin were proposed responsible for the biocontrol activity of *B. atrophaeus* GQJK17 [81]. *B. methylotrophicus* B25, which enhances plant health, carries several genes responsible for secondary metabolism, such as nonribosomal peptide synthetases and polyketide synthases [82], and *Paenibacillus polymyxa* strains SC2 and E681 possess genes required for biosynthesis of antibiotics such as polymyxin and fusaricidin [83,84]. The genome of one of the best-known biocontrol strains, *P. fluorescens* Pf-5, was shown diverse with secondary metabolism genes [85,86], and another *Pseudomonas* biocontrol strain, SH-C52, was reported equally versatile with biosynthesis of secondary metabolites, such as thanamycin, hydrogen cyanide, achromobactin- and ornicorrugatin-type siderophores, bacteriocin, arylpolyene, insect toxins Tcc2 and Tcc4, and three NRPS-based lipopeptides 2 [87]. Most recently, reports on the genomes of potential biocontrol strains *Streptomyces* sp. JBS5-6 and *B. toyonensis* BAC3151 listed similarly gene clusters responsible of secondary metabolite biosynthesis [88,89]. Common traits found in sequenced *B. polymyxa* strains with plant-growth-promoting ability are phytohormone production and increased nutrient availability [90]. In the genome of the biofertilizer *Rhodopseudomonas palustris* ELI 1980, genes for antibiotic production, auxin biosynthesis, and nitrogen fixation were identified [91]. Newly reported *Sphingomonas* sp. Cra possesses a number of genes related to plant-growth promotion, a trait which was confirmed for *Arabidopsis thaliana* [92].

Regardless of the high potential, analysis of genomes has rarely revealed new traits behind plant-growth promotion or protection against biotic or abiotic stresses. There are a couple of examples, however. In the biofertilizer strain *Trichoderma harzianum* t-22, genome mining revealed biosynthesis of tricholignan A, which is a natural product helping the plant in assimilating iron from the soil [93]. In the plant-growth-promoting strain *Methylorubrum extorquens* DSM13060, which increases pine growth to the same extent as mycorrhizal fungi, genome analysis confirmed lack of plant hormone biosynthesis genes, but revealed an array of phospholipase A genes, potentially responsible for growth promotion [94]. In *Micrococcus luteus* K39, phospholipase D, superoxide dismutase, and ferredoxin NADP reductase were suggested responsible for induced tolerance of oxidative stress, salinity, and drought in the host [95]. A recent comparative analysis of an array of plant-associated bacterial genomes revealed 64 plant-resembling domains, including NLR class of intracellular innate immune receptors, which were hypothesized to interfere with plant immune functions. Such intracellular immune receptors could potentially be responsible of enhancing plant resistance by biocontrol strains as a new trait [96].

### 5.2. Molecular Plant–Microbe Interactions

A detailed analysis of interactions between the host plant and the microbe can reveal new beneficial traits in biofertilizers and biocontrol agents. For example, a study on gene expression of *B. velezensis* FZB42 suggests the involvement of regulatory small RNAs in the plant–microbe interaction [97]. A detailed study on the beneficial compounds produced by the plant-growth-promoting strain *M. extorquens* DSM13060 revealed that bacterial metabolism of polyhydroxybutyrate plays an important role in stress tolerance of the bacterium and host [98,99]. In the biocontrol strain *P. fluorescens* Pf0-1, the importance of histidine metabolism during the interaction with host plants has been discovered through gene-expression analyses [100]. Furthermore, antimicrobial peptides are a group of less studied, but potentially important compounds associated with biocontrol, which have been discovered through studies on bioactive metabolites in both fungal [101] and bacterial endophytes [102,103].

### 5.3. Microbe–Microbe Interactions

Another largely unknown field is the microbe–microbe interactions, and studies on such interactions would likely reveal completely new traits useful in biocontrol and biofertilization. Once a microbe is inoculated into a plant, it affects the full holobiont, the host, and the associated microbiome. This is clearly seen with pathogens infecting host plants, as they create shifts in the structure of endophytic communities [104,105]. Similar but less pronounced changes have been observed as the result of endophyte inoculation in potato [106,107]. An example of microbe–microbe interactions as a mechanism in biocontrol is the endophytic community playing a role in control of citrus variegated chlorosis (CVC) by *Methylobacterium mesophilicum* in sweet orange (*Citrus sinensis* L.). The pathogen *Xylella fastidiosa* invades the xylem vessels of sweet orange, where the colonization is suppressed by resident endophytic community influenced by *M. mesophilicum* [108]. This suggests that the performance of specific biocontrol agents can in reality be the consequence of a community response. Further information on microbe–microbe interactions is necessary for a full understanding of the action of biofertilizers and biocontrol agents. Therefore, analysis of the microbial community of the holobiont should accompany studies on beneficial effects of microorganisms in plants.

### 5.4. Phage Interactions

Bacteriophages, or phages, are emerging as a new trait in biocontrol, replacing or accompanying bacterial biocontrol agents [109,110]. Phage interactions are typical for a root microbiome [111], which suggests phages could be useful tools in biocontrol. For a successful tailored product, life cycle and host interactions of the pathogen need to be thoroughly understood [110]. The antimicrobial mechanisms are based on lysis of bacterial cells caused by virulent phages or lytic enzymes. The phages could also be manipulated genetically to deliver specific lethal genes to the target organisms, or to sensitize them against traditional antimicrobials [109] which, however, raises the problems of spreading antibiotic resistance and responsible usage of genetically modified organisms (GMOs) in the environment. Overall, phages may represent an “outside of the box” solution in biocontrol, and such unconventional thinking is necessary to create new innovations in agriculture.

## 6. How to Identify New Potent Microbial Strains

A current and widely approved trend involves using multiple (2–5) microbiological tools in combination to achieve more effective and consistent results in agriculture. Plant-growth promotion can be enhanced by using several biofertilizer strains, both fungal and bacterial, together, complementing the effects of each other [112,113]. Similarly, microorganisms with biocontrol capacities can have a synergetic effect and stimulate the antagonism of other biocontrol agents against plant diseases [18]. However, when *B. velezensis* FZB42 was studied in the rhizosphere, the main mechanism of protecting the host plant was not the production of antibiotic compounds by single or several strains in combination, but the systemic resistance induced by the bacteria in the host plant [114]. These results emphasize the importance of a healthy microbiome, which is behind the healthy phenotype in animals [115], as well as in plants [116]. For example, a recent study showed that the composition of soil microbiome could predict the survival of a plant host against disease. Specifically, species belonging to genera *Pseudomonas* and *Bacillus* were important members of the protective microbiome [116]. Microbiome engineering, i.e., rationally constructing microbiomes and inoculating them into crop plants could be a solution for sustainable agriculture of the future. Such attempts should not only focus on the root microbiome, but on the full plant microbiome including shoot endophytic communities [117]. However, how to find new bacterial and fungal strains as strategic members of a healthy microbiome?

### 6.1. Modification of Microbial Genomes

Genomic modification of microorganisms can provide more potent strains of biofertilizers or biocontrol tools. Ideas and attempts to genetically enhance biocontrol agents have already been cultivated and tested [118,119]. For example, *P. syringae* was modified early on to enhance frost tolerance of strawberry and potato by removing ice nucleation protein from the bacterial genome [120]. *P. fluorescens* strains F113 and CHA0, as well as *P. putida* WCS358 have been genetically engineered for enhanced production of antibiotic compounds, 4-diacetylphloroglucinol (PHL) and pyoluteorin (PLT). Furthermore, a strain of *P. fluorescens* CHA0 has been modified to overproduce the phytohormone indole acetic acid (IAA). However, when tested in a microcosm, the *P. fluorescens* strain CHA0 overproducing PHL and PLT decreased the numbers of rhizobia, *Sinorhizobium meliloti*, and reduced nodulation in alphalpha. The IAA-overproducing strain of CHA0 increased root yield in natural soil, but reduced root growth in autoclaved soil [121]. The development of genome editing using CRISPR/Cas system has revived the field of using genetically modified microorganisms to fight plant diseases [122,123]. For example, development of non-pathogenic strains of fungal pathogens by CRISPR/Cas was recently proposed as an approach of biocontrol [124]. However, fungal endophytes that are close, non-pathogenic relatives of plant pathogens are already typically an innate part of plant microbiomes [125], therefore, the approach is already naturally in use.

Overall, there are severe ethical aspects associated with GMOs as biofertilizers or biocontrol agents in agriculture [122]. Some tests with genetically enhanced biocontrol agents have shown changes in the microbial community structure of the rhizosphere [121]. More importantly, the GMOs would be released to the environment and, the transferred traits, such as biosynthesis genes of antimicrobial compounds, would with a very high likelihood spread across microbiomes, spreading antimicrobial resistance. Furthermore, restricting the access of the GMOs to the edible plant parts would need considerate testing. A deliberate release of genetically modified organisms into the environment is regulated by Council Directive 2001/18/EC, which repeals Council Directive 90/220/EEC in Europe [121,122]. Nevertheless, genetic modification using CRISPR/Cas, for example, is an important tool in elucidating the mechanisms of plant–microbe interaction [123].

### 6.2. Bacterial Endophytes and Endosymbionts

Apart from the PGPR that can have an endophytic phase, or rhizobia of legumes, bacterial endophytes have rarely been the choice for biocontrol or biofertilizer products. However, the currently used microbiological tools that typically colonize plant rhizosphere, soil, root surface, or root apoplast, have demonstrated inconsistent field performances, especially when used as single inoculations [63]. Systemic bacterial endophytes, which colonize all internal tissues within a plant, and endosymbionts, which colonize plant cells, would likely provide more persistent benefits to the host plant than rhizobacteria or apoplastic endophytes. They would be better protected from environmental conditions, such as abiotic stress, being well adapted to the targeted niche [63,94,106,107,126]. Endophytic bacteria share many growth-promoting and biocontrol traits with PGPR, such as production of plant hormones and antibiotic compounds, induction of systemic resistance, or nitrogen fixation [117,127,128,129].

**Figure 2:**
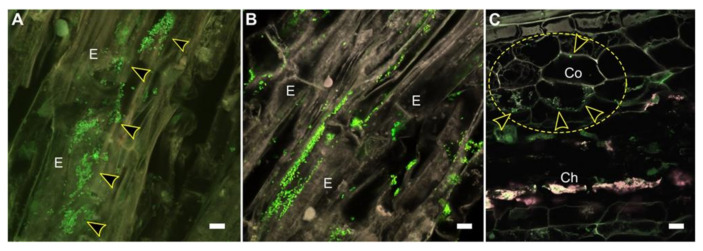
Confocal laser scanning microscopy of Scots pine seedlings colonized by *M. extorquens* 13060. Bacteria colonizing the pine tissues are carrying a Green Fluorescent Protein (GFP) reporter under a constitutive promoter and visualized in bright green. (**A**) Lateral section of a pine root 30 days post-inoculation (dpi). Arrowheads indicate *M. extorquens* cells on the root epidermis. (**B**) Individual bacterial cells are visible in the root cortex. (**B**) Lateral section of a pine root 50 dpi where bacteria are colonizing epidermal cells. (**C**) Cross-section of a pine root from a higher region 60 dpi with intracellular bacteria depicted by arrowheads in the cortical cells (circled). Co, cortex; Ch, chlorenchyma; E, epiderm; scale bars, 10 µm.

Potentially limiting the use of shoot endophytes in agriculture is their existence in the edible parts of the crop plant [63]. However, this line of product development should be reconsidered. The fear of introducing microorganisms into edible plant parts has no basis on the grounds of the fact that plant tissues are already naturally colonized by a variety of microorganisms, both completely harmless strains, as well as potential human pathogens [117,130]. Selection of non-pathogenic microorganisms should pose no threat to human health, which can be ascertained through food security programs. Furthermore, several food products, such as fermented foods, are already manufactured with the help of an array of microorganisms, and they are considered healthy for the human gut [131]. The shoot endophytic strains could be applied to host plants during micropropagation, as many crop plants are routinely propagated in vitro. For crops propagated through seeds, formulations similar to those prepared for rhizobacteria could provide a suitable application method. For example, the endosymbiont *M. extorquens* DSM13060 systematically colonizes the host plant after application of bacterial cells on germinating roots (Figure 2) [94].

### 6.3. Microorganisms of Plants Adapted to Extreme Growth Conditions

So far, the majority of biofertilizers and biocontrol agents have been identified from rhizosphere of crop plants. For example, among the PGPR, *Azotobacter chroococcum* has been isolated from wheat rhizosphere [132], *Azospirillum brasilense* strains from rice and wheat [133], and *Pseudomonas fluorescens* Pf5 from rhizosphere of cotton seedlings [134]. Although some strains can be host-specific, many are generalists, and plants growing in extreme growth conditions are fruitful targets for finding new potent biofertilizers and biocontrol agents, because they often rely on their microbial partners in surviving in the challenging environment [135]. For example, the growth-promoting endosymbiont *M. extorquens* DSM13060 has been isolated from Scots pine (*Pinus sylvestris* L.), which is a conifer species adapted to harsh growing conditions, poor levels of nutrients in the soil, drought, and low temperatures [136]. The endophytic fungus *Curvularia protuberata* provides heat resistance in the tropical panic grass *Dichanthelium lanuginosum*, which can be transferred to crop species such as tomato, watermelon, and wheat [137]. Similarly, *Piriformospora indica*, isolated from a desert plant, provides tolerance towards salt and biotic stress in several crop plants [46,47,48]. New effective biocontrol agents are also likely to be discovered in resistant plants living in environments with a high pathogen reservoir (for example, see Reference [138]).

### 6.4. Microorganisms of Diverse Environments

When searching for new biofertilizers and biocontrol agents, a rational choice is to screen environments with high microbial diversity. With soils harboring the greatest reservoirs of microbes, land-use change is considered the most severe threat to biodiversity. A recent study showed that conversion of forest soils to arable land changes especially bacterial diversity, but also viral diversity, whereas Archaean diversity was less responsive. The bacterial diversity was reduced by 45–75%, depending on forest type, and correlated with loss of functional diversity in the soil [139]. There are similar reports on fungal endophytes. In a forest setting, the diversity of foliar fungal endophytes declines along a gradient of pine trees growing in old-growth forest, managed forest, and in nursery [140]. Grapevine varieties grown on organically managed farms host richer fungal endophyte communities than the varieties grown under an integrated pest-management system [141]. The bacterial endophytic communities respond in a similar manner to biodiversity of the environment. For example, using a green manure by *Erythrina poeppigiana* (Walp.) O. F. Cook or *Inga* trees modifies the endophytic microbiome of banana, compared to plants grown in monoculture [142,143]. Therefore, environments rich with various plant species, such as forests, likely host a rich pool of microorganisms beneficial for plants. Discovery of new microbial strains that can be used as members of an engineered healthy plant microbiome, or as single inoculants in biocontrol and biofertilization, is more fruitful in forests than on agricultural land.

## 7. Conclusions and Future Prospects

The earth is currently suffering from chemical pollution on all fronts. To ensure that the next generations have a healthy living environment, effective solutions must be found and executed promptly. Further development and increased use of biofertilizers and biocontrol agents is necessary for sustainable food production in the future. Currently, we have ~50 functional strains that are applied in agriculture (Figure 1 and Supplementary Table S1). The demand for both biofertilizers and biocontrol agents is expected to rise, which provides a good basis for further development.

The major problem regarding the current tools is the inconsistency of the products in field conditions. Using combinations of microbial strains that rely on different biocontrol or biofertilizer properties could alleviate the problem of inconsistency; this approach is already is in use to some extent. However, while the current tools are all based on the few well-known mechanisms of action, plant–microbe and microbe–microbe interactions are more multifaceted than that and need a thorough investigation. Detailed understanding of each microbial strain, their mechanisms of action, and components of a healthy plant microbiome will likely provide a firm basis for developing reliable tools to enhance plant health and growth in varying field conditions. To achieve this, the following are needed:

(1) A continuous search for new efficient microorganisms, enabling healthy plant cultivation in various field conditions, is pertinent. The potential new microbiological tools are likely to be found in plants adapted to extreme, in nutrient-poor growth conditions, in resistant plants under pathogen exposure, and in environments with high biodiversity, such as forests.
(2) Studies on microbial traits beneficial for plants should be extended beyond mechanisms already known. The research for new microbial traits should cover not only plant–microbe interactions, but also microbe–microbe interactions, including phage effects and transmission.
(3) The focus should be turned from the rhizosphere towards the plant holobiont, including strains occupying all plant organs, both bacteria and fungi. A high number of microbial inoculants should be combined in the biocontrol and biofertilizer products, with the aim of plant microbiome engineering.
(4) For enabling the production of such complex biological products, the bottleneck of formulation needs to be solved. Therefore, more efforts should be placed on the development of technologies for microbial culture and live preservation. With the recent enormous advances in molecular and microbiological techniques, a technological jump enabling microbial live preservation in various conditions should be more than feasible.
(5) A thorough analysis of the capacity of the strains to enhance plant health and growth under several growth conditions should become a routine in biofertilizer and biocontrol agent discovery. There are frequent reports in the scientific literature on new potent strains identified, but usually they are only tested in the laboratory conditions for few of the well-known microbial traits, and in one or two plant-growth conditions [144,145]. The effects of inoculants need to be tested under stressful conditions, along with an analysis of changes in the microbiome of the holobiont.

To conclude, a full array of well-studied essential microbiome components in a formulation easily applied on crops could provide reliable, consistent results of crop production in any field condition of future agriculture.

## Data Availability

Not applicable.

## Supplementary Materials

The following are available online at https://www.mdpi.com/article/10.3390/microorganisms9040817/s1, Table S1: Microbiologicaltools inagriculture, available on the markets in 2021 with published field test reports.
