# Supplementary material for: Biofertilizers and Biocontrol Agents for Agriculture: How to Identify and Develop New Potent Microbial Strains and Traits
Source: Microorganisms. 2021 Apr 13;9(4):817. doi: 10.3390/microorganisms9040817 (PMC8069042; doi:10.3390/microorganisms9040817)
Supplement: Supplementary file 1 [file microorganisms-09-00817-s001.pdf]

Table S1. Microbiological tools in agriculture, available on the markets in 2021 with published field test reports.

Name	Species	Organism type	Application	Target	Reference
Amylo-X	*Bacillus amyloliquefaciens*	PGPR	biocontrol	Controls gray mold (*Botrytis cinerea*), Fire blight (*Erwinia amylovora*) in grapes, apple,pPear, strawberry	Ladurner, E., Benuzzi, M., Fiorentini, F., & Lucchi, A. (2012). Efficacy of Amylo-X®, a new fungicide/bactericide based on *Bacillus amyloliquefaciens* strain D747 for the control of microbial plant pathogens. Giornate Fitopatologiche 2012, Milano Marittima (RA), 13-16 marzo 2012, 229-236.
ANOKA	*Trichoderma viride, Pseudomonas fluorescens*	mix	biocontrol	Control of damping-off, seed and root, nematode wilt in vegetable, tuber, cotton, chilli crops	Woo, S. L., Ruocco, M., Vinale, F., Nigro, M., Marra, R., Lombardi, N., ... & Lorito, M. (2014). *Trichoderma*-based products and their widespread use in agriculture. The Open Mycology Journal, 8(1).
AQ10 Biofungicide	*Ampelomyces quisqalis* M-10	soil fungi	biocontrol	Controls powdery mildews in grapes, apples, aubergine, tomatoes, strawberry, cucumber	Shishkoff, N., & McGrath, M. T. (2002). AQ10 biofungicide combined with chemical fungicides or AddQ spray adjuvant for control of cucurbit powdery mildew in detached leaf culture. Plant Disease, 86(8), 915-918.
Azotovit	*Azotobacter chroococcum*	PGPR	biofertilizer	Improves nitrogen availability in maize and broccoli	Alafeea, R. A. A., Alamery, A. A., & Kalaf, I. T. (2019). Effect of bio fertilizers on increasing the efficiency of using chemical fertilizers on the yield component of maize (*Zea mays* L.). Plant Archives, 19(2), 303-306.
BactoFil A10	*Azospirillum brasilense, Azotobacter vinelandii, Bacillus megaterium, B. polymyxa, Pseudomonas fluorescens*	mix	biofertilizer	Improves nutrient acquisition on ryegrass	Katai, J., Sandor, Z., & Tallai, M. (2008). The effect of an artificial and a bacterium fertilizer on some soil characteristics and on the biomass of the rye-grass (*Lolium perenne* L.). Cereal Research Communications, 36, 1171-1174.
BactoFil B10	*Azospirillum brasilense, Azotobacter vinelandii, Bacillus*	mix	biofertilizer	Provides higher yield of secondary metabolites in anise, coriander	Aćimović, M. G., Dolijanović, Z. K., Oljača, S. A., Kovačević, D. D., &

	megaterium, B. circulans, B. subtilis, Pseudomonas fluorescens				Oljača, M. V. (2015). Effect of organic and mineral fertilizers on essential oil content in Caraway, Anise and Coriander fruits. Acta Sci. Pol., Hortorum Cultus, 14(1), 95-103.
Binab T	*Trichoderma harzianum, T. polysporum*	epiphytic fungi	biocontrol	Biofungicide against soil, foliar and vascular pathogens use greenhouses, nurseries, horticultural, fruits, trees and ornamental crops	Woo, S. L., Ruocco, M., Vinale, F., Nigro, M., Marra, R., Lombardi, N., ... & Lorito, M. (2014). *Trichoderma*-based products and their widespread use in agriculture. The Open Mycology Journal, 8(1).
Biofox C	*Fusarium oxysporum*	soil fungi	biocontrol	Against root pathogens tomato, cucumber, pepper, monocot and dicot species	Minuto, A., Migheli, Q., & Garibaldi, A. (1995). Evaluation of antagonistic strains of *Fusarium* spp. in the biological and integrated control of *Fusarium* wilt of cyclamen. Crop Protection, 14(3), 221-226.
Bio-P	*Bacillus megaterium*	PGPR	biofertilizer	Improves phosphorus acquisition in tomato	Youssef, M. A., & Eissa, M. A. (2017). Comparison between organic and inorganic nutrition for tomato. Journal of Plant Nutrition, 40(13), 1900-1907.
Bio-K	*Frateuria aurantia*	PGPR	biofertilizer	Improves potassium acquisition in tomato	Youssef, M. A., & Eissa, M. A. (2017). Comparison between organic and inorganic nutrition for tomato. Journal of Plant Nutrition, 40(13), 1900-1907.
Bio-N	*Azotobacter* sp.	PGPR	biofertilizer	Improves nitrogen acquisition in tomato	Youssef, M. A., & Eissa, M. A. (2017). Comparison between organic and inorganic nutrition for tomato. Journal of Plant Nutrition, 40(13), 1900-1907.
Biomonas	*Pseudomonas fluorescens*	PGPR	biocontrol	Against damping-off of chilli caused by *Pythium aphanidermatum*	Saha, S., Rai, A. B., & Pandey, S. (2011). Efficacy of seed dressing agents against damping-off disease of chilli (*Capsicum frutescens*). Indian Journal of Agricultural Sciences, 81(1), 92.
Bio-save 10LP	*Pseudomonas syringae* ESC-10, ESC-11	PGPR	biocontrol	Against postharvest diseases in apples, pears, lemons, oranges, or grapefruit	Bettiol W, Morandi MAB, Pinto ZV, de Paula Jr TJ, Corrêa EB, Moura AB, Lucon CMM, Costa JCB, Bezerra JL

					(2012) Produtos comerciais à base de agentes de biocontrole de doenças de plantas, Jaguariúna, SP: Embrapa Meio Ambiente
Bio-Ject Spot-Less	*Pseudomonas aureofaciens strain* Tx-1	PGPR	biocontrol	Controls dollar spot, anthracnose, pythium, pink snow mold in turfgrass	Fravel, D. R. (2005). Commercialization and implementation of biocontrol. Annu. Rev. Phytopathol., 43, 337-359.
Biogro	*Pseudomonas fluorescens, Candida tropicalis, Bacillus amyloliquefaciens, Bacillus subtilis*	mix	biofertilizer	Nitrogen fixation, phosphorous solubilization, organic matter mineralization	Hien, N. T., Toan, P. V., Choudhury, A. T., Rose, M. T., Roughley, R. J., & Kennedy, I. R. (2014). Field application strategies for the inoculant biofertilizer BioGro supplementing fertilizer nitrogen application in rice production. Journal of Plant Nutrition, 37(11), 1837-1858.
BioYield	*Bacillus subtilis* GB03, *Bacillus amyloliquefaciens* IN937a	PGPR	biofertilizer	Increases root and shoot mass, stem caliper, and the root to shoot ratio of tomato, pepper, cucumber, tobacco, and melons	Kloepper, J. W., Gutierrez-Estrada, A., & McInroy, J. A. (2007). Photoperiod regulates elicitation of growth promotion but not induced resistance by plant growth-promoting rhizobacteria. Canadian Journal of Microbiology, 53(2), 159-167.
Blossom-Protect, Boni-Protect	*Aureobasidium pullulans* DSM 14940 (CF 10), DSM 14941 (CF 40)	epiphytic fungi	biocontrol	Developed and registered to control postharvest diseases of apple as the product	Weiss A, Mögel G (2006). Kunz S Development of “Boni-Protect”- a yeast preparation for use in the control of post-harvest diseases of apples. In: Boos M (ed) 12th International conference on cultivation technique and phytopathologtical problems in organic fruit-growing. Weinsberg, Germany, pp 113–117
Blight Ban A506	*Pseudomonas fluorescens* A506	PGPR	biocontrol	Against blight in fruits, tomato, potato	Sharifazizi, M., Harighi, B., & Sadeghi, A. (2017). Evaluation of biological control of *Erwinia amylovora*, causal agent of fire blight disease of pear by antagonistic bacteria. Biological Control, 104, 28-34.
Botrycid	*Burkholderia vietnamiensis*	PGPR	biocontrol	Controls soil fungal pathogens, plant-pathogenic bacteria, and nematodes	Harman, G. E., Obregón, M. A., Samuels, G. J., & Lorito, M. (2010).

					Changing models for commercialization and implementation of biocontrol in the developing and the developed world. Plant Disease, 94(8), 928-939.
Botrybel	*Bacillus velezensis*	PGPR	biocontrol	Controls gray mold (*Botrytis cinerea*)	Fernández, A., Villaverde, M., Casanova, J., Malo, J., Nicolas, J. A., & Blanca, I. (2004). Nuevo aislado de *Bacillus* y su utilización para el control de hongos fitopatógenos. Agroecologia. net, 6, 50-57.
CataPult SuperFine	*Glomus intraradices, Bacillus* spp.	mix	biofertilizer	Phosphorous solubilization and mineralization, drought resistance in cereals, tree and vine crops	Mishra, J., & Arora, N. K. (2016). Bioformulations for plant growth promotion and combating phytopathogens: a sustainable approach. In Bioformulations: For sustainable agriculture (pp. 3-33). Springer, New Delhi.
Cedomon, Cedress	*Pseudomonas chlororapsis*	PGPR	biocontrol	Against leaf stripe, net blotch, *Fusarium* spp., spot blotch, leaf spot in barley and oats	Mehnaz, D., Wang, Q., Gross, H., Li, Y., Jahanshah, G., & Paterson, J. (2016). The contribution of genome mining strategies to the understanding of active principles of PGPR strains.
Ecomonas, Florezen P	*Pseudomonas fluorescens*	PGPR	biocontrol	Controls sheath blight (*Rhizoctonia solani*) on rice	Kumar, K. V. K., Raju, S. K., Reddy, M. S., Kloepper, J. W., Lawrence, K. S., Groth, D. E., ... & Binghai, D. (2009). Evaluation of commercially available PGPR for control of rice sheath blight caused by *Rhizoctonia solani.* J Pure Appl Microbiol, 3(2), 485-488.
Ecosom-Th	*Trichoderma harzianum*	epiphytic fungi	biocontrol	Controls *Sclerotinia sclerotiorum* on mustard (*Brassica juncea*)	Yadav, M. S., Godika, S., Yadava, D. K., Ahmad, N., Mehta, N., Bhatnagar, K., ... & Chattopadhyay, C. (2019). Prioritizing components of package of integrated pest management in Indian mustard (*Brassica juncea*) in India for better economic benefit. Crop Protection, 120, 21-29.
Flocter WP5	*Bacillus firmus*	PGPR	biocontrol	Controls root-knot nematodes	Catalkaya, M., & DEVRAN, Z. (2019). Integrated management of Mi-

					1 virulent *Meloidogyne incognita* (Kofoid & White, 1919) Chitwood, 1949 (Tylenchida: Meloidogynidae) in greenhouse tomatoes. Türkiye Entomoloji Dergisi, 43(2), 157-169.
FZB24	*Bacillus amyloliquefaciens* ssp. *plantarum*	PGPR	biofertilizer	Ornamentals, vegetables, field crops	Gul, A., Kidoglu, F., Tüzel, Y., & Tüzel, I. H. (2008). Effects of nutrition and" *Bacillus amyloliquefaciens*" on tomato (" *Solanum lycopersicum* L.") growing in perlite. Spanish Journal of Agricultural Research, (3), 422-429.
Gmax PGPR	*Azotobacter, Pseudomonas fluorescens*	mix	biofertilizer	Field crops	Hafeez, F. Y., Abaid-Ullah, M., & Hassan, M. N. (2013). Plant growth-promoting rhizobacteria as zinc mobilizers: a promising approach for cereals biofortification. In Bacteria in agrobiology: crop productivity (pp. 217-235). Springer, Berlin, Heidelberg.
Jumpstart	*Penicillium bilaie*	soil fungi	biofertilizer	Helps the plant in phosphate acquisition	Leggett, M., Cross, J., Hnatowich, G., & Holloway, G. (2007). Challenges in commercializing a phosphate-solubilizing microorganism: *Penicillium bilaiae*, a case history. In First international meeting on microbial phosphate solubilization (pp. 215-222). Springer, Dordrecht.
Ketomium	*Chaetomium cupreum*	soil fungi	biocontrol	Biofungicide against pathogens. Used in maize, potato	Soytong, K., Kanokmedhakul, S., Kukongviriyapa, V., & Isobe, M. (2001). Application of *Chaetomium* species (Ketomium) as a new broad spectrum biological fungicide for plant disease control. Fungal Divers, 7, 1-15.
Koni, Cotans WG	*Coniothyrium minitans*	soil fungi	biocontrol	Against Pathogens *Sclerotinia*, Registered for soybeans, canola, sunflower, safflower, dry edible beans, lettuce, carrots, snap beans, cabbage, tomato and celery	Butt, T. M., & Copping, L. G. (2000). Fungal biological control agents. Pesticide Outlook, 11(5), 186-191.

Met52	*Metarhizium brunneum*	entomopathogenic fungi	biocontrol	Effective in reducing the abundance of *Ixodes scapularis*, the tick	Fischhoff, I. R., Keesing, F., & Ostfeld, R. S. (2017). The tick biocontrol agent *Metarhizium brunneum* (= *M. anisopliae*) (strain F52) does not reduce non-target arthropods. PloS one, 12(11), e0187675.
Micofert	*Glomus claroideum, Acaulospora kentinensis, Diversispora spurca, Glomus etunicatum*	mycorrhiza	biofertilizer	Improves nutrient availability in lettuce	Ley-Rivas, J. F., Aliagar, L., Moron, C., & Furrazola-Gómez, E. (2011). Efecto del biofertilizante MICOFERT en la producción de dos variedades de lechuga en Perú. Acta Botánica Cubana, (213), 36-39.
Micosat F Uno	*Agrobacterium radiobacter* AR 39*, Bacillus subtilis* BA 41*, Streptomyces spp.* SB 14*, Glomus spp.* GB 67*, Funneliformis mosseae* GP 11*, G. viscosum* GC 41*, Pochonia chlamydosporia* PC 50 e*, Trichoderma harzianum* TH 01*, Pichia pastoris* PP 59	mix	biofertilizer	Fruits, vegetables, flowers	Iacono, F., Conte, G., Giovannetti, G., Longo, V., & Porro, D. (2010). Esperienze in vigneto sull'uso delle micorrize. III Convegno nazionale di viticoltura: libro dei riassunti.
Myc 800	*Rhizophagus intraradices*	mycorrhiza	biofertilizer	Improves nutrient acquisition in strawberry	Mikiciuk, G., Sas-Paszt, L., Mikiciuk, M., Derkowska, E., Trzciński, P., Głuszek, S., ... & Rudnicka, J. (2019). Mycorrhizal frequency, physiological parameters, and yield of strawberry plants inoculated with endomycorrhizal fungi and rhizosphere bacteria. Mycorrhiza, 29(5), 489-501.
MycoApply Endo, VAM 80	*Glomus intraradices*	mycorrhiza	biofertilizer	Improves phosphate availability in corn	Corkidi, L., Allen, E. B., Merhaut, D., Allen, M. F., Downer, J., Bohn, J., & Evans, M. (2004). Assessing the infectivity of commercial mycorrhizal inoculants in plant nursery conditions. Journal of Environmental Horticulture, 22(3), 149-154.
Mycostop	*Streptomyces griseoviridis* K61	PGPR	biocontrol	Controls many fungal pathogens, such as *Fusarium oxysporum* f.sp. lycopersici and *Verticillium dahliae*	Minuto, A., Spadaro, D., Garibaldi, A., & Gullino, M. L. (2006). Control of soilborne pathogens of tomato

					using a commercial formulation of *Streptomyces griseoviridis* and solarization. Crop Protection, 25(5), 468-475.
MycoTrol	*Beauveria bassiana* GHA	entomopathogenic fungi	biocontrol	Controls potato beetle larvae	Wraight, S. P., & Ramos, M. E. (2017). Characterization of the synergistic interaction between *Beauveria bassiana* strain GHA and *Bacillus thuringiensis morrisoni* strain tenebrionis applied against Colorado potato beetle larvae. Journal of invertebrate pathology, 144, 47-57.
MycoUp, Resid	*Glomus iranicum* var. *tenuihypharum*	mycorrhiza	biofertilizer	Improves phosphate availability in leaf vegetables, berries, fruit, olives, grapes, greenhouse crops and cereals.	Martín, F. F., Molina, J. J., Nicolás, E. N., Alarcón, J. J., Kirchmair, M., García, F. J., ... & Bernal, C. (2017). Application of Arbuscular Mycorrhizae *Glomus iranicum* var. tenuihypharum var. nova in Intensive Agriculture: a study case. J. Agric. Sci. Technol. B, 7, 221-247.
Mykoflor	*Rhizophagus irregularis, Funneliformis mosseae, Claroideoglomus etunicatum*	mycorrhiza	biofertilizer	Improves nutrient acquisition in strawberry	Mikiciuk, G., Sas-Paszt, L., Mikiciuk, M., Derkowska, E., Trzciński, P., Głuszek, S., ... & Rudnicka, J. (2019). Mycorrhizal frequency, physiological parameters, and yield of strawberry plants inoculated with endomycorrhizal fungi and rhizosphere bacteria. Mycorrhiza, 29(5), 489-501.
Nexy	*Candida oleophila* I-182	epiphytic fungi	biocontrol	Against a postharvest diseases, strain O has been approved as a plant protection agent in Europe in 2013 (European Commission Health & Consumers Directorate-General 2013; European Food Safety Authority (EFSA) 2015a)	Wisniewski, M., Wilson, C., Droby, S., Chalutz, E., El-Ghaouth, A., & Stevens, C. (2007). Postharvest biocontrol: new concepts and applications. Biological control: A global perspective, 262-273.
Nitragin Gold	*Rhizobium meliloti*	rhizobia	biofertilizer	Provides nitrogen fixation for alfalfa, sweet clover or clover	Smith, R. S. (1995). Inoculant formulations and applications to meet changing needs. In Nitrogen fixation: fundamentals and applications (pp. 653-657). Springer, Dordrecht.

Nitroguard	*Azospirillum brasilense* NAB317*, Azorhizobium caulinodens* NAB38*, Azoarcus indigens* NAB04*, Bacillus* sp.	mix	biofertilizer	Nitrogen fixation, phosphate solubilization on cereals, seed rape, sugar beet, sugarcane, vegetables	Le Mire, G., Nguyen, M., Fassotte, B., du Jardin, P., Verheggen, F., Delaplace, P., & Jijakli, H. (2016). Implementing biostimulants and biocontrol strategies in the agroecological management of cultivated ecosystems. Biotechnologie, Agronomie, Société et Environnement.
Nitrofix	*Azospirillum* str. Az39	PGPR	biofertilizer	Imrpoves nitrogen acquisition	Okon, Y., Labandera-Gonzales, C., Lage, M., & Lage, P. (2015). Agronomic applications of *Azospirillum* and other PGPR. Biological nitrogen fixation, 921-933.
Noli	*Metschnikowia fructicola* NRRL Y-27328	epiphytic fungi	biocontrol	A plant protection agent against fungal diseases in stone fruits, strawberries and grapes	Kurtzman, C. P., & Droby, S. (2001). *Metschnikowia fructicola*, a new ascosporic yeast with potential for biocontrol of postharvest fruit rots. Systematic and applied microbiology, 24(3), 395-399.
Novodor	*Bacillus thuringiensis morrisoni*	entomopathogeni c bacterium	biocontrol	Controls potato beetle larvae	Wraight, S. P., & Ramos, M. E. (2017). Characterization of the synergistic interaction between *Beauveria bassiana* strain GHA and *Bacillus thuringiensis morrisoni* strain tenebrionis applied against Colorado potato beetle larvae. Journal of invertebrate pathology, 144, 47-57.
Phylazonit-M	*Pseudomonas putida, Azotobacter chroococcum, Bacillus circulans, Bacillus megaterium*	mix	biofertilizer	Improves phosphate availability in rice, maize	Ingle, K. P., & Padole, D. A. (2017). Phosphate solubilizing microbes: an overview. Int J Curr Microbiol Appl Sci, 6(1), 844-852.
Proradix	*Pseudomonas sp.* DSMZ 13134	PGPR	biocontrol	Produces organic and siderophore acids, highly effective in chelating metal cations (zinc, copper and iron) in potato, tomato, cucumber, paprika, zucchini, grass	Fröhlich, A., Buddrus-Schiemann, K., Durner, J., Hartmann, A., & Von Rad, U. (2012). Response of barley to root colonization by *Pseudomonas* sp. DSMZ 13134 under laboratory, greenhouse, and field conditions. Journal of Plant Interactions, 7(1), 1-9.
Rhizocare	*Trichoderma viride*	epiphytic fungi	biocontrol	Controls wilts and rots diseases on crops	Tanwar, A., Aggarwal, A., & Panwar, V. (2013). Arbuscular mycorrhizal

					fungi and *Trichoderma viride* mediated *Fusarium* wilt control in tomato. Biocontrol Science and Technology, 23(5), 485-498.
Rhizocell	*Bacillus amyloliquefaciens* IT45	PGPR	biofertilizer	Phosphorous solubilization and mineralization in vegetables, horticultural and field crops	Xie, L., Lehvävirta, S., Timonen, S., Kasurinen, J., Niemikapee, J., & Valkonen, J. P. (2018). Species-specific synergistic effects of two plant growth—promoting microbes on green roof plant biomass and photosynthetic efficiency. PloS one, 13(12), e0209432.
RhizoMyco, RhizoMyx, RhizoPlex	A mixture of 18 species of endo- and ectomycorrhizal fungi	mycorrhiza	biofertilizer	Improves nutrient and water availability in several crops	Poddar, R., Sen, A., Kundu, R., Das, H., & Bandopadhyay, P. (2020). Response of Various Mycorrhizal Inoculants on Rice Growth, Productivity and Nutrient Uptake. International Journal of Bio-resource and Stress Management.
Rhizosum N	*Azotobacter vinelandi, Rhizophagus irregularis*	mix	biofertilizer	Improves nitrogen and phosphate availability in several crops	Dal Cortivo, C., Ferrari, M., Visioli, G., Lauro, M., Fornasier, F., Barion, G., ... & Vamerali, T. (2020). Effects of seed-applied biofertilizers on rhizosphere biodiversity and growth of common wheat (*Triticum aestivum* L.) in the field. Frontiers in plant science, 11, 72.
Rhizosum PK	*Bacillus megaterium, Rhizophagus irregularis, Frateuria aurantia*	mix	biofertilizer	Improves phosphate and potassium availability in several crops	Dal Cortivo, C., Ferrari, M., Visioli, G., Lauro, M., Fornasier, F., Barion, G., ... & Vamerali, T. (2020). Effects of seed-applied biofertilizers on rhizosphere biodiversity and growth of common wheat (*Triticum aestivum* L.) in the field. Frontiers in plant science, 11, 72.
Rhizovital 42	*Bacillus amyloliquefaciens*	PGPR	biofertilizer	Vegetables, horticultural and field crops	Aćimović, M., Jaćimović, G., Oljača, S., Sharaf-Eldin, M., Đukanović, L., & Vuga-Janjatović, V. (2011). Efficasy (ie Efficacy) of biofertilizers on seed germination and yield of caraway,

					anise and coriander. Annales of Scientific Work.
Rizofos Liq Maíz	*Pseudomonas fluorescens*	PGPR	biofertilizer	Phosphorous solubilization and mineralization, production of phytohormones, siderophore and antibiotics in maize, wheat	Deambrosi, E., Méndez, R., & Avila, S. (2004). Evaluación de efectos del uso de rizofos en el cultivo de arroz. INIA Serie Actividades de Difusión.
Romeo, Cerevisane	*Saccharomyces cerevisiae*	epiphytic fungi	biocontrol	Preventive inducer of systemic resistance against powdery and downy mildew in grapes, fruits and vegetables	De Miccolis Angelini, R. M., Rotolo, C., Gerin, D., Abate, D., Pollastro, S., & Faretra, F. (2019). Global transcriptome analysis and differentially expressed genes in grapevine after application of the yeast-derived defense inducer cerevisane. Pest management science, 75(7), 2020-2033.
Rotstop S	*Phlebiopsis gigantea*	saprophyte	biocontrol	Pathogen *Heterobasidion annosum* in conifer trees	Sun, H., Paulin, L., Alatalo, E., & Asiegbu, F. O. (2011). Response of living tissues of *Pinus sylvestris* to the saprotrophic biocontrol fungus *Phlebiopsis gigantea*. Tree Physiology, 31(4), 438–451
Serenade	*Bacillus subtilis*	PGPR	biocontrol	Against *Botrytis* bunch rot, brown rot, Powdery mildew (grapes, apple, tomato, onion, citrus, peach, pear)	Matheron, M. E., & Porchas, M. (2000). Evaluation of fungicide performance for control of powdery mildew on lettuce in 2000.
SoilGard	*Gliocladium virens*	soil fungi	biocontrol	Controls damping-off of zinnia, cotton, and cabbage caused by *Pythium ultimum* or *Rhizoctonia solani* in nonsterile soilless mix	Lumsden, R. D., & Walter, J. F. (1996). Development of *Gliocladium virens* for damping-off disease control. Canadian Journal of Plant Pathology, 18(4), 463-468.
Sonata	*Bacillus pumilus* QST 2028	PGPR	biocontrol	Against powdery mildew, downy mildew, blight, and soyabean rust (grapes, strawberry, pome fruits)	Borriss, R. (2011). Use of plant-associated *Bacillus* strains as biofertilizers and biocontrol agents in agriculture. In Bacteria in agrobiology: Plant growth responses (pp. 41-76). Springer, Berlin, Heidelberg.
Suma Grow	14 bacterial *(Rhizobium spp., Pseudomonas spp., Bacillus spp.)* and 7 fungal species *(Trichoderma spp.)*	mix	biofertilizer	Improves nutrient acquisition in several crops (corn, sorghum, rice, pea, okra, peanut, pea, beans, squash, tomato, clover)	Janarthanam, L. (2013). Bioprotectant with multifunctional microorganisms: A new dimension in plant protection. Journal of Biopesticides, 6(2), 219.

Symbion-N	*Azospirillum, Rhizobium, Acetobacter, Azotobacter*	mix	biofertilizer	Nitrogen fixation on field crops, vegetables	Abd El Ghafour, A., Darwish, M. A., Azoz, S. N., Abd-Alla, A. M., & Elsayed, S. I. (2017). Effect of mineral, bio and organic fertilizers on productivity, essential oil composition and fruit anatomy of two dill cultivars (Anethum graveolens L.). Sciences, 7(03), 532-550.
Symbion-P	*Bacillus megaterium* var. *phosphaticum*	PGPR	biofertilizer	Phosphorous solubilization and mineralization in vegetables, horticultural and field crops	Abd El Ghafour, A., Darwish, M. A., Azoz, S. N., Abd-Alla, A. M., & Elsayed, S. I. (2017). Effect of mineral, bio and organic fertilizers on productivity, essential oil composition and fruit anatomy of two dill cultivars (*Anethum graveolens* L.). Sciences, 7(03), 532-550.
Symbion-K	*Frateuria aurantia*	PGPR	biofertilizer	Potassium solubilization on horticultural and field crops	Abd El Ghafour, A., Darwish, M. A., Azoz, S. N., Abd-Alla, A. M., & Elsayed, S. I. (2017). Effect of mineral, bio and organic fertilizers on productivity, essential oil composition and fruit anatomy of two dill cultivars (*Anethum graveolens* L.). Sciences, 7(03), 532-550.
Tagteam	*Penicillium bilaii, Rhizobium leguminosarum*	mix	biofertilizer	Improves nitrogen availability in cowpea	Vaishnavi, S. J., & Jeyakumar, P. (2016). Bioinoculant on microbial population, biochemical characters and yield of cowpea. Environment and Ecology, 34(1), 129-131.
Taegro	*Bacillus subtilis*	PGPR	biocontrol	Controls fungal diseases on apple	Nasir, M., Idress, M., Iqbal, B., Hussain, M., Mohy-ud-Din, G., & Ayub, M. (2017). Comparative efficacy of biocontrol agent Bacillus subtilis and fungicides against powdery mildew of apple. J. Agric. Res, 55(1), 75-84.
Trichoderma x	*Trichoderma asperellum*	soil fungi	biocontrol	Controls soil fungal pathogens on seeds, roots and flowers in horticultural crops	Woo, S. L., Ruocco, M., Vinale, F., Nigro, M., Marra, R., Lombardi, N., ... & Lorito, M. (2014). *Trichoderma*-based products and their widespread

					use in agriculture. The Open Mycology Journal, 8(1).
Twin N	*Azospirillum brasilense* NAB317*, Azorhizobium caulinodens* NAB38*, Azoarcus indigens* NAB0	mix	biofertilizer	Nitrogen fixation on cereals, seed rape, sugar beet, sugarcane, vegetables	Le Mire, G., Nguyen, M., Fassotte, B., du Jardin, P., Verheggen, F., Delaplace, P., & Jijakli, H. (2016). Implementing biostimulants and biocontrol strategies in the agroecological management of cultivated ecosystems. Biotechnologie, Agronomie, Société et Environnement.
